# Fibroblast activation protein is expressed by rheumatoid myofibroblast-like synoviocytes

**DOI:** 10.1186/ar2080

**Published:** 2006-11-14

**Authors:** Stefan Bauer, Michael C Jendro, Andreas Wadle, Sascha Kleber, Frank Stenner, Robert Dinser, Anja Reich, Erica Faccin, Stefan Gödde, Harald Dinges, Ulf Müller-Ladner, Christoph Renner

**Affiliations:** 1 Oncology Department, UniversitätsSpital Zürich, Rämistrasse 100, 8091 Zürich, Switzerland; 2 Med. Department I, Universität des Saarlandes, Kirrbergstrasse, 66421 Homburg/Saar, Germany; 3 Department of Internal Medicine and Rheumatology, University of Giessen and Kerckhoff-Clinic, Benekestrasse 2–8, 61231 Bad Nauheim, Germany; 4 Orthopedic Department, Universität des Saarlandes, Kirrbergstrasse, 66421 Homburg/Saar, Germany; 5 Orthopedic Clinic, Westpfalz-Klinikum, Im Flur 1, 66869 Kusel, Germany

## Abstract

Fibroblast activation protein (FAP), as described so far, is a type II cell surface serine protease expressed by fibroblastic cells in areas of active tissue remodelling such as tumour stroma or healing wounds. We investigated the expression of FAP by fibroblast-like synoviocytes (FLSs) and compared the synovial expression pattern in rheumatoid arthritis (RA) and osteoarthritis (OA) patients. Synovial tissue from diseased joints of 20 patients, 10 patients with refractory RA and 10 patients with end-stage OA, was collected during routine surgery. As a result, FLSs from intensively inflamed synovial tissues of refractory RA expressed FAP at high density. Moreover, FAP expression was co-localised with matrix metalloproteinases (MMP-1 and MMP-13) and CD44 splice variants v3 and v7/8 known to play a major role in the concert of extracellular matrix degradation. The pattern of signals appeared to constitute a characteristic feature of FLSs involved in rheumatoid arthritic joint-destructive processes. These FAP-expressing FLSs with a phenotype of smooth muscle actin-positive myofibroblasts were located in the lining layer of the synovium and differ distinctly from Thy-1-expressing and non-proliferating fibroblasts of the articular matrix. The intensity of FAP-specific staining in synovial tissue from patients with RA was found to be different when compared with end-stage OA. Because expression of FAP by RA FLSs has not been described before, the findings of this study highlight a novel element in cartilage and bone destruction of arthritic joints. Moreover, the specific expression pattern qualifies FAP as a therapeutic target for inhibiting the destructive potential of fibroblast-like synovial cells.

## Introduction

Fibroblast activation protein (FAP) is an *M*_r _95-kDa, cell surface-bound, type II transmembrane glycoprotein and belongs to the family of serine prolyl oligopeptidases. Comparison of amino acid sequences indicates that FAP is essentially identical to seprase [1] and closely related to dipeptidylpeptidase IV (DPP IV), also known as CD26, another type II integral membrane protein [2]. These exoproteases cleave NH_2_-terminal dipeptides from polypeptides with l-proline or l-alanine in the penultimate position. In addition, FAP was found to bear collagenase activity *in vitro *[1,3]. Peptidase activity of FAP, in addition to various families of proteolytic enzymes such as matrix or disintegrin metalloproteases that serve as major collagenases, contributes to extracellular matrix (ECM) degradation [4,5]. This not only is a fundamental property of normal tissue repair and remodelling but also is involved in the pathological processes of invasive growth. It correlates with the expression of FAP in granulation tissue of healing wounds and in more than 90% of human epithelial carcinomas [6]. Consistent with its mesenchymal origin, FAP is also occasionally expressed by bone and soft tissue sarcomas [7]. Immunohistochemical staining of colorectal carcinomas and breast cancer [4,8] confirmed the specific expression of FAP by tumour stroma fibroblasts but not by malignant cells themselves. In contrast, resting fibrocytes in normal adult tissue generally lack detectable FAP expression [7].

Rheumatoid arthritis (RA) is a chronic inflammatory disease of unknown aetiology and is characterised by hyperplasia and chronic inflammation of the synovial membranes that invade deeply into the articular cartilage and bone. Activated fibroblast-like synoviocytes (FLSs) in the lining layer of the synovium are among the dominant cell types involved in pannus formation and are key players in joint destruction [9,10]. Rheumatoid FLSs have been shown to proliferate in an anchorage-independent manner and express increased proliferation markers and matrix-degrading enzymes when compared with FLSs from patients with osteoarthritis (OA) [11-13]. Expression of the CD44v7/8 epitopes contributes to the proliferative behaviour of FLSs obtained from patients with RA, whereas expression of variants containing v3 is linked with their increased invasive capacity [14-16]. Matrix metalloproteases (MMPs) have been shown to be essential for degradation of articular matrix, with MMP-1 and MMP-13 being important candidates for joint destruction in RA [17,18]. However, except for metallocollagenolytic activities, invasion of migratory fibroblasts into connective tissue involves serine types of cell surface proteases [5]. Among the exoproteases that may cooperate with interstitial collagenase are groups of serine prolyl peptidases, including DPP IV/CD26 and FAP/seprase [4,19]. Gene expression signatures of FLSs demonstrate the association of a high inflammatory state of synovitis and the presence of a myofibroblast-like molecular phenotype [20]. These myofibroblasts were identified in cultures from patients with RA [21] as well as by immunohistochemical staining in the intimal lining layer of RA synovial tissues [20].

Here, we report the concomitant involvement of FAP together with metalloproteases and CD44 variants in the lining layer of rheumatoid synovium contributing to the characteristics of FLSs with myofibroblastic phenotype. In addition, synovial tissue analysis revealed a strong correlation between inflammatory synovitis and FAP expression.

## Materials and methods

### Patients

Synovial tissues were collected during routine orthopaedic surgery from 10 consecutive patients with end-stage OA and 10 consecutive patients with refractory destructive RA who underwent joint replacement. The latter patient population fulfilled the American College of Rheumatology criteria for RA [22]. The mean age of patients with RA was 62 years (eight females and two males). Mean laboratory parameters were erythrocyte sedimentation rate of 41 mm/hour and leucocyte count of 8.95 × 10^3 ^per microlitre, and 90% of patients were rheumatoid factor-positive. All patients gave informed consent, and the study protocol was approved by the local ethics committees.

### Cell lines and reagents

RPMI 1640 medium (supplemented with 10% [vol/vol] heat-inactivated foetal calf serum, penicillin [100 units per millilitre], streptomycin [0.1 mg/ml], and glutamine [0.3 mg/ml]) (all from Gibco, now part of Invitrogen Corporation, Carlsbad, CA, USA) was used as standard medium. HT1080 FAP-transfected (HT1080 FAP) and mock-transfected (HT1080 par) cells were previously described [23]. The following antibodies were obtained from the following sources: Murine F19 (mouse anti-human FAP monoclonal antibody) was obtained from the Ludwig Institute for Cancer Research (New York, NY, USA). Anti-human CD90 (Thy-1; clone AS02) and biotin-SP-conjugated rabbit anti-mouse immunoglobulin G (IgG) were purchased from Dianova GmbH (Hamburg, Germany). Mouse anti-human CD44v3 (clone VFF-327) and CD44v7/8 (clone VFF-17) were purchased from Serotec Ltd. (Oxford, UK), and mouse anti-human smooth muscle actin (SMA) (clone 1A4) was obtained from Dako Denmark A/S (Glostrup, Denmark). Goat anti-human MMP-1 (C-18) and MMP-13 (D-17) were purchased from Santa Cruz Biotechnology, Inc. (Santa Cruz, CA, USA). Biotinylated rabbit anti-goat IgG was purchased from Vector Laboratories (Burlingame, CA, USA).

### RNA isolation and reverse transcription-assisted polymerase chain reaction

FAP- or mock-transfected HT1080 cells, tumour samples from patients with breast cancer, or a part of collected synovial tissue was homogenised immediately after surgery by using a tissue tearer, and total RNA was extracted (RNeasy; Qiagen GmbH, Hilden, Germany) following the manufacturer's instructions. Total amount of RNA was quantified by spectrophotometry, and 500 ng was used for first-strand cDNA synthesis using RevertAid™ H Kit (Fermentas GmbH, St. Leon-Rot, Germany). The following primers were used for amplification of specific cDNA fragments as described previously [24]: The FAP polymerase chain reaction (PCR) product was generated with 5'-GTTATTGCCTATTCCTATTATG-3' and 5'-GTCCATCATGAAGGGTGGAAA-3'. The MMP-1 PCR product was generated with 5'-CTGAAGGTGATGAAGCAGCC-3' and 5'-AGTCCAAGAGAATGGCCGAG-3'. The MMP-13 PCR product was generated with 5'-CTATGGTCCAGGAGATGAAG-3' and 5'-AGAGTCTTGCCTGTATCCTC-3'. The GADPH (glyceraldehyde-3-phosphate dehydrogenase) PCR product was generated with 5'-GTGAAGGTCGGAGTCAACGGATTT-3' and 5'-CTCCTTGGAGGCCATGTGGGCCAT-3'. cDNA was amplified with 1.25 units of Taq DNA polymerase with 2.5 ml of 10× standard taq buffer (Thermopol; New England Biolabs, Ipswich, MA, USA) in a final volume of 25 μl containing 0.5 μl of nucleosidtriphosphate (10 mM), 0.5 μl of each primer (10 μM), and Aqua dest. PCR amplification was carried out on a PTC-200 thermocycler (MJ Research, now part of Bio-Rad, Hercules, CA, USA) with the following profile: 95°C for 5 minutes, annealing for 60 seconds, and extension at 72°C for 1 minute. The number of cycles and the annealing temperature depended on the primers used: (a) FAP (55°C, 30 cycles), (b) MMP-1, (c) MMP-13 (62°C, 40 cycles), and (d) GADPH (62°C, 25 cycles). Aliquots (20 μl) of each PCR product were separated on a 1.5% agarose gel and visualised by ethidium bromide staining (Sigma-Aldrich, St. Louis, MO, USA). Cell lines or breast cancer samples served as controls.

### Quantitative real-time PCR

Measurement was performed on LightCycler detection systems (Roche, Heidelberg, Germany) using the relative quantification method with external standard (LightCycler technical note no. LC 10/update 2003; Roche). The endogenous control gene hypoxanthine phosphoribosyltransferase 1 (*HPRT1*; NM_000194) and the *FAP *gene (NM_004460) were measured by quantitative reverse transcription-PCR using the GenGlobe™ technology from Qiagen GmbH and an HPRT quantification kit (Roche). Design of specific HPRT and human FAP primers was executed by Qiagen GmbH (QuantiTect Primer Assay, QT00074963) and Roche. Assay conditions for the real-time PCR were adjusted using an optimised PCR protocol with the following profile: initial activating of Taq-polymerase at 95°C for 15 minutes, followed by 35 cycles with denaturation at 95°C for 15 seconds, annealing at 60°C for 15 seconds, and extension at 72°C for 20 seconds. Samples from all patients (10 patients with RA and 10 patients with OA), controls, and standards were run in triplicate. The amount of the housekeeping gene *HRPT *was used to correlate *FAP *expression employing LightCycler analysis software.

### Immunohistochemistry

Remaining portions of collected samples were embedded in Tissue-Tek OCT medium (Miles Diagnostics, Elkhart, IN, USA), snap-frozen in liquid nitrogen, and then stored at -80°C for immunohistochemical analysis. Sequential 5-μm sections were placed on SuperFrost^®^Plus microscope slides (Menzel-Gläser, Braunschweig, Germany). Slides were fixed in cold acetone (4°C for 10 minutes), air-dried, rehydrated in phosphate-buffered saline, and then blocked using a Biotin Blocking System (Dako North America, Inc., Carpinteria, CA, USA). Before the primary antibody (anti-human FAP, anti-human Thy-1, or irrelevant isotype-matched IgG, 1:200, incubated for 60 minutes at room temperature; anti-human CD44v3, 1:50; anti-human CD44v7/8, 1:200; anti-human SMA, 1:50; and anti-human MMP-1, anti-human MMP-13, or irrelevant isotype-matched IgG, 1:100, incubated overnight at 4°C) was added, slides were blocked with rabbit serum from the Vectastain^® ^ABC Kit (Vector Laboratories). The biotinylated secondary antibody (rabbit anti-mouse IgG [1:500] or rabbit anti-goat IgG [2 μg/ml]) and the preformed avidin-biotinylated horseradish peroxidase P-complex (ABC reagent) were used according to the manufacturer's instructions. The antibody-ABC complex was visualised with a 3-amino-9 ethylcarbazole (AEC) (Sigma-Aldrich)-based chromagen, resulting in a pink-brown colouration of antigen-positive cells. Simultaneous staining of FAP and SMA was performed using the DakoCytomation EnVision Doublestain-Kit (code K1395; Dako North America, Inc.) according to the manufacturer's instructions. FAP was stained first with DAB (3,3'-diaminobenzidin) followed by colouration of SMA with fast red. All slides were counterstained for approximately 2 to 5 minutes with Meyer's haematoxylin. Final slide adjustment was performed using Adobe Photoshop Elements (Adobe Systems GmbH, Unterschleissheim, Munich, Germany).

### Statistics

Comparison of *FAP *gene expression measured by real-time PCR between synovial tissue samples taken from patients with end-stage OA or severe RA was performed with Mann-Whitney test (*U *test). *P *values < 0.01 were considered statistically significant.

## Results

### FAP mRNA expression and protein transcription profile

**Figure 1:**
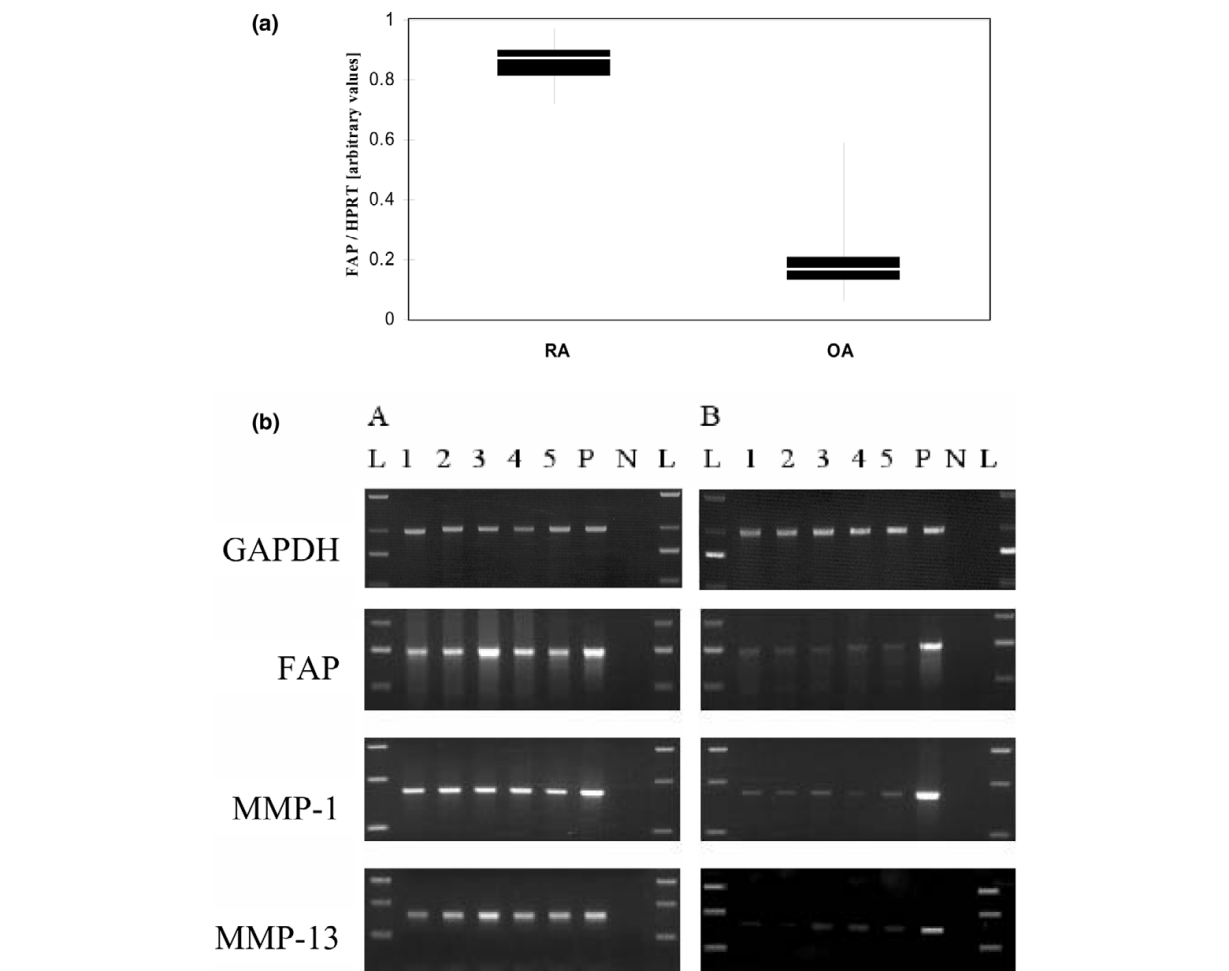
Expression of fibroblast activation protein (FAP) mRNA. Synovial tissue samples were collected by orthopaedic surgery from 10 patients with end-stage osteoarthritis (OA) who underwent joint replacement or from 10 patients with destructive refractory rheumatoid arthritis (RA). **(a) **Quantification of *FAP *gene expression was determined by real-time polymerase chain reaction (PCR) as described in Materials and methods. The data are presented relative to *HPRT *and are graphically visualised as box-and-whisker plots. Expression of *FAP *gene is significantly higher in the synovial samples of patients with RA compared with OA synovial samples (*p *< 0.01). **(b) **Reverse transcription-PCR analysis of FAP, matrix metalloproteinase (MMP)-1, and MMP-13. Synovial tissue samples were collected from patients with destructive refractory RA (column A, lanes 1 to 5) or from patients with end-stage OA (column B, lanes 1 to 5). Extraction control was performed with GAPDH PCR. Positive controls (P) were fibroblasts for MMP-1, pooled breast cancer samples for amplification of MMP-13 and p53, and FAP-transfected HT1080 cells for FAP. GAPDH, glyceraldehyde-3-phosphate dehydrogenase; HPRT, hypoxanthine phosphoribosyltransferase; L, DNA ladder; N, negative controls.

**Figure 2:**
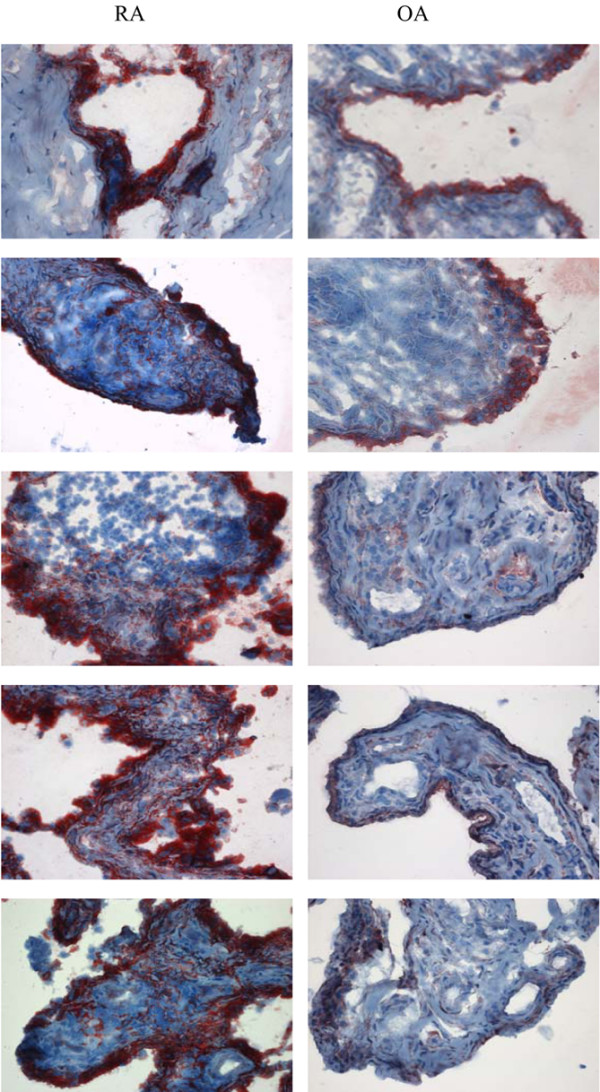
Cell surface expression of fibroblast activation protein (FAP). Gene expression analysis is completed by immunohistochemical staining. FAP-specific staining was performed on synovial samples of five individuals of each entity (rheumatoid arthritis [RA] and osteoarthritis [OA]), demonstrating the stronger expression at the protein level in the inflamed synovia of patients with refractory RA. Magnification, ×100.

Synovial tissue was collected from 20 patients, 10 patients with refractory RA and 10 patients with end-stage OA. mRNA extraction and cDNA synthesis were performed immediately after surgery. Quantitative real-time PCR analysis was established to compare the levels of *FAP *gene expression in synovial samples of both entities. *FAP *gene expression was significantly higher in RA synovial samples (*p *< 0.01; Figure 1a). High specificity of PCR is demonstrated by agarose gel electrophoresis and melting curve analysis (data not shown). Furthermore, all samples were analysed for mRNA transcripts coding for fragments of FAP (741 bp), MMP-1 (428 bp), and MMP-13 (390 bp). Amplification of a GADPH fragment with the expected size of 995 bp served as control. Positive PCR controls were obtained from cDNA derived from FAP-transfected HT1080 cells (FAP) or breast cancer tumour samples (MMP-1 and MMP-13). Amplification of cDNA derived from mock-transfected HT1080 cells was negative for specific fragments. Analysis of synovial samples from patients with severe RA (Figure 1b, column A) or end-stage OA (Figure 1b, column B) demonstrates ubiquitary expression of MMP-1, MMP-13, and FAP. However, expression was more pronounced in refractory RA samples. Differences in transcription of *FAP *gene were confirmed by immunohistochemistry. Corresponding to the significant difference in gene expression analysis, FAP positivity is much more pronounced in rheumatoid synovial samples than in the osteoarthritic counterparts (Figure 2).

### Association of FAP with activation markers

**Figure 3:**
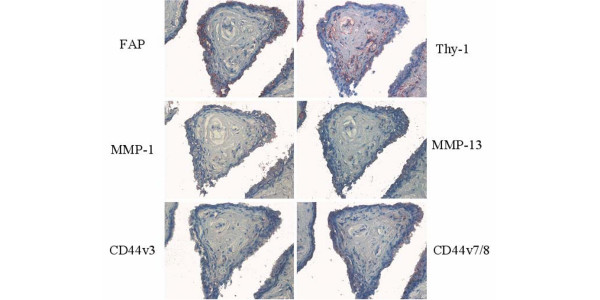
Immunohistochemical analysis of activation markers in synovial membranes of osteoarthritis. Phenotypic markers that are instrumental in extracellular matrix alteration are detectable only at a low level (MMP-1, CD44v7/8) in areas that are slightly FAP-positive. Magnification, ×400. FAP, fibroblast activation protein; MMP, matrix metalloproteinase.

**Figure 4:**
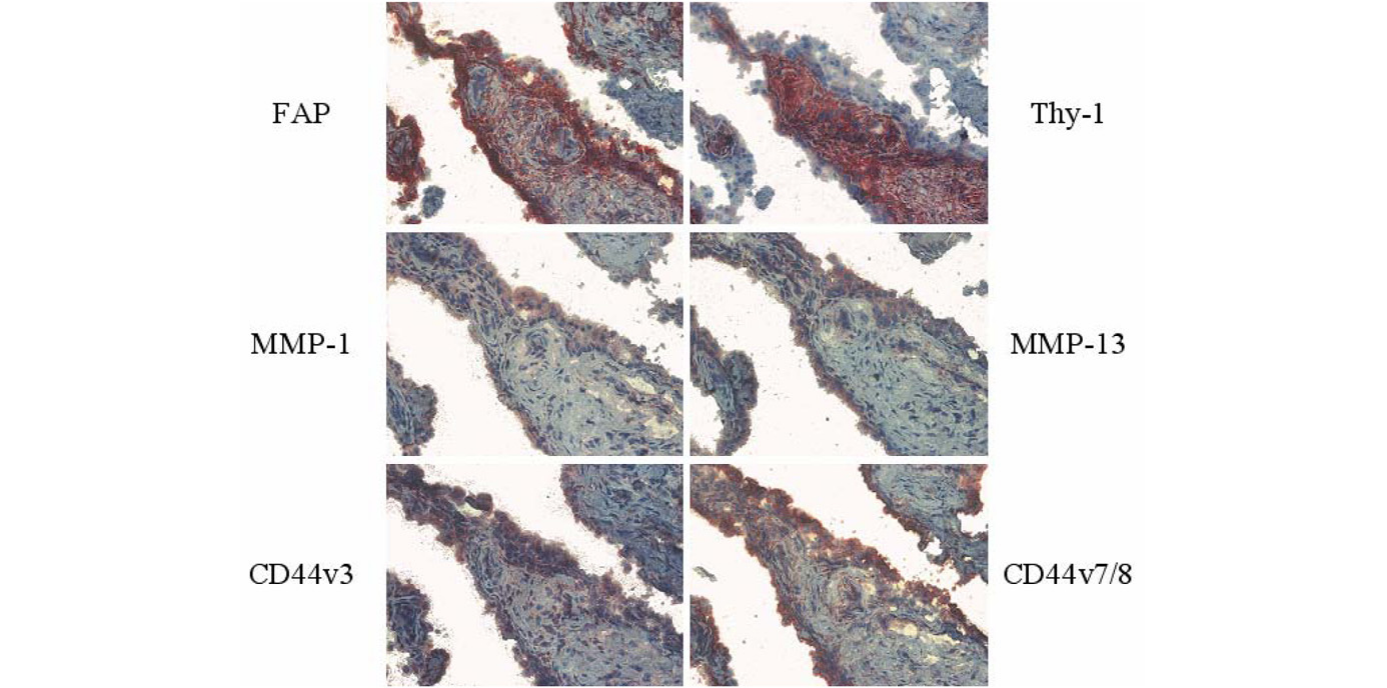
Immunohistochemical analysis of activation markers in synovial membranes of rheumatoid arthritis. Expression of fibroblast activation protein (FAP) is accompanied by accumulation of activation markers (MMP-1 and MMP-13) and splice variants of CD44 (v3 and v7/8) that are known to correlate with the degree of inflammation and that are involved in extracellular matrix deposition. Magnification, ×400. MMP, matrix metalloproteinase.

**Figure 5:**
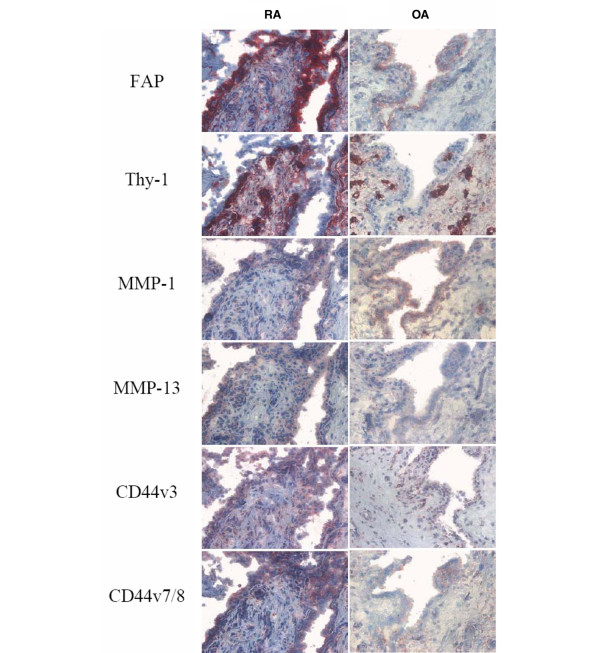
Comparison of immunohistochemical detection of activation markers. Analysis of representative areas of synovial tissue samples taken from patients with end-stage osteoarthritis (OA) or refractory rheumatoid arthritis (RA) is shown. Expression pattern and staining intensity of RA samples represent the stronger intensity of synovial inflammation when compared with OA samples. Magnification, ×400. FAP, fibroblast activation protein; MMP, matrix metalloproteinase.

The histomorphological characterisation of the synovial lining layer by FAP expression is accompanied by the accumulation of other activation markers in this area. The phenotypic markers analysed in this context were MMP-1 and MMP-13, together with the v3 and v7/8 splice variants of CD44, which are instrumental in ECM alteration in malignancies and already known to be expressed in the synovial membranes of diseased joints (Figures 3 and 4). The expression signature characterises the area of FAP-expressing cells as the centre of high inflammatory activity in the rheumatoid synovium (Figures 4 and 5). In contrast to the synovial lining layer of rheumatoid joints, osteoarthritic joints show limited staining for MMPs and CD44 variants, with only minor expression of MMP-13 and CD44v7/8 (Figures 3 and 5).

### Association of FAP with fibroblast markers

**Figure 6:**
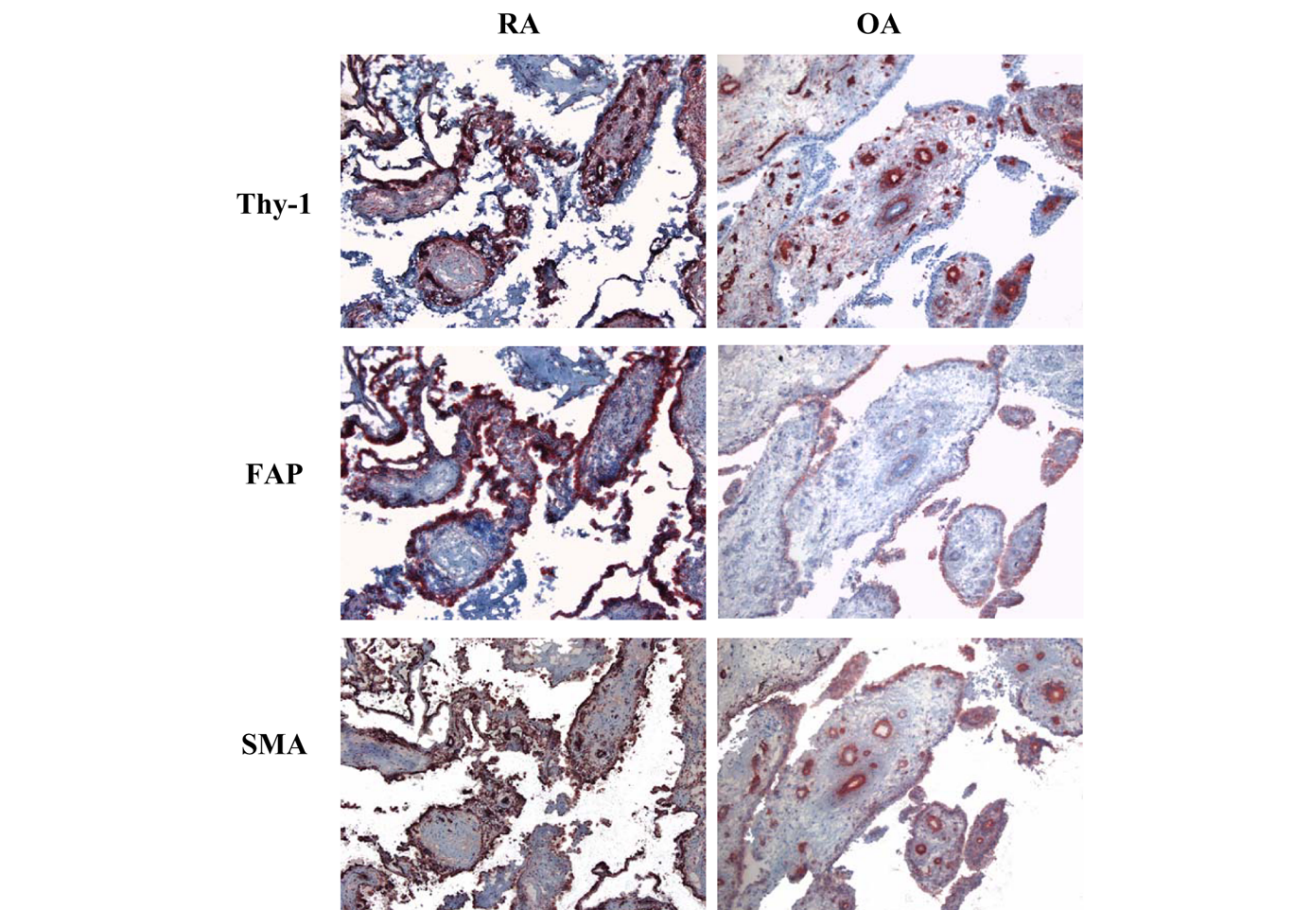
Immunohistochemical characterisation of fibroblast populations. Collected samples were snap-frozen, and sequential sections were stained for expression of Thy-1, fibroblast activation protein (FAP), or smooth muscle actin (SMA). Antigen-positive cells were identified by pink-brown colouration. Tissue samples of both entities, rheumatoid arthritis (RA) and osteoarthritis (OA), show distinct synovial fibroblast populations. Associated expression of SMA and FAP distinguishes myofibroblasts of the intimal synovial lining from Thy-1-positive fibroblasts or only SMA-expressing perivascular smooth muscle cells. Staining of anti-human FAP and anti-human SMA is more intense in RA tissue samples when compared with OA. Magnification, ×100.

**Figure 7:**
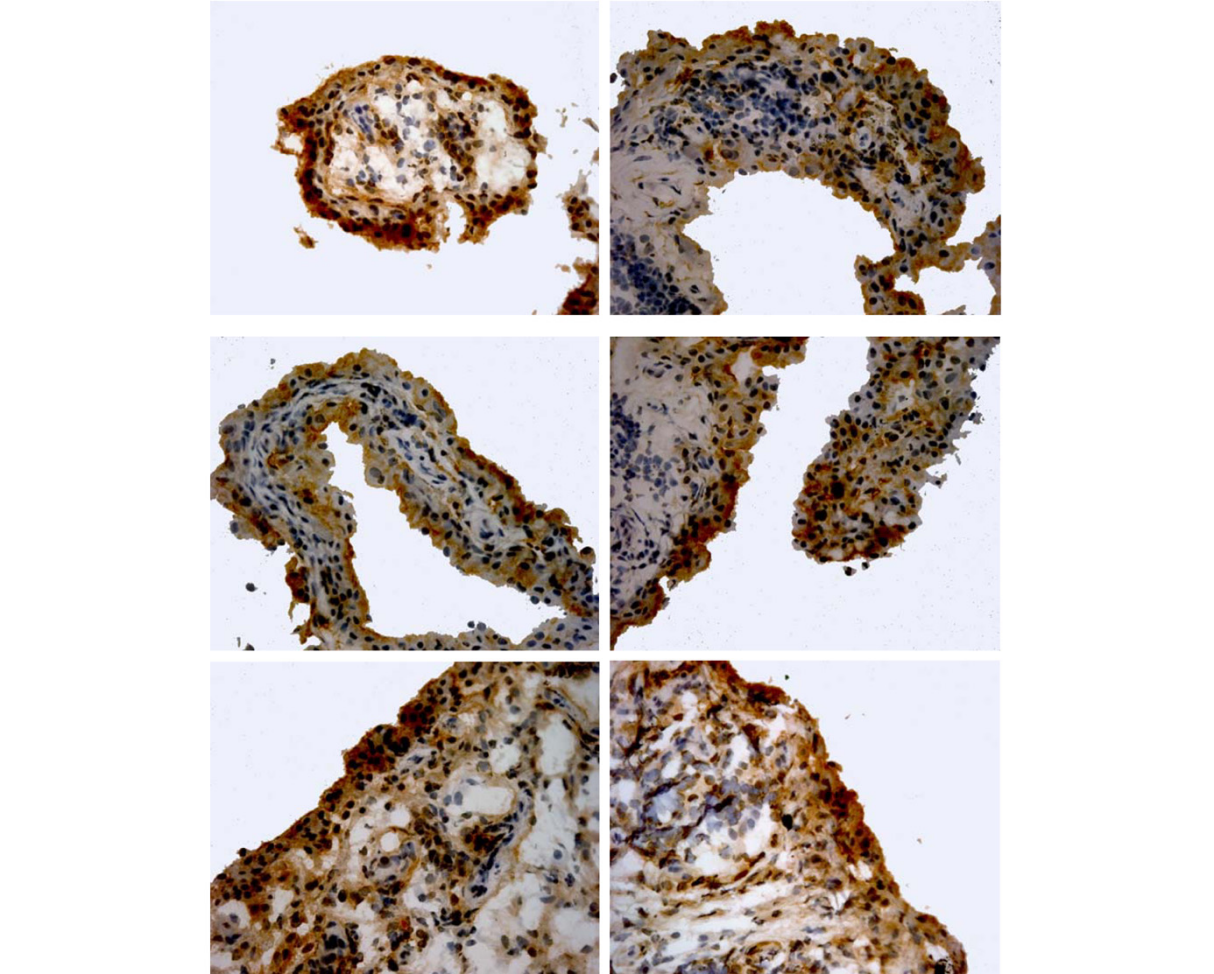
Double-staining of fibroblast activation protein (FAP) and smooth muscle actin (SMA). Synovial tissue samples from patients with destructive rheumatoid arthritis were stained to demonstrate the simultaneous expression of both antigens. FAP-positive fibroblasts in the intimal synovial lining were coloured brown (DAB [3,3'-diaminobenzidin]). Coexpression of SMA is visualised in red (fast red). Magnification, ×400.

RA and OA synovial fibroblasts express CD90 (Thy-1) on their surface. However, there is a distinct fibroblast population in the lining layer of the synovium from both patient groups, completely lacking Thy-1 surface expression. This distinct synovial cell population is clearly characterised by cell surface expression of FAP (Figure 6) which is paralleled by cell surface expression of activation markers (Figure 5). Fibroblasts with myofibroblastic phenotype are a known source of proteolytic enzymes that are involved in ECM deposition. A reliable marker for identifying this subpopulation is anti-SMA. In addition to staining of perivascular smooth muscle cells, immunohistochemistry revealed the expression of SMA by FAP-positive FLSs in the intimal lining layer (Figure 6), and the simultaneous expression of both antigens could be demonstrated by double-staining technique (Figure 7). As a result, expression pattern and staining intensity of SMA are considerably different between the rheumatoid and the osteoarthritic synovium. Furthermore, either the expression density of activation markers or their staining intensity differs between the samples of osteoarthritic synovial tissue from different patients. With regard to the degree of inflammation, immunohistochemical analysis of synovial samples from patients with refractory RA showed homogenous expression pattern and staining intensity.

## Discussion

The main finding of our study is the demonstration that FAP is expressed by synovial fibroblasts in patients with RA and OA. It is well established that FAP is expressed mainly on the surface of mesenchymal cells that are involved in epithelium-mesenchyme interactions contributing to tissue remodelling. So far, FAP-expressing fibroblasts in human have been detected only in granulation tissue of healing wounds [25], desmoplastic reactions [26], and the reactive stroma of malignant tissue [27]. Resting fibrocytes in normal adult tissue generally lack expression of FAP [6]. However, observations from a clinical Phase I study with a humanised anti-FAP antibody might retrospectively lead to the conclusion that FAP could also be present in human joints. Studying the biodistribution of a humanised anti-FAP antibody in patients with advanced or metastatic FAP-positive cancer, a minor low-grade uptake in the knees and shoulders of three patients without clinical symptoms of arthritis was observed. Unfortunately, no further explanation or discussion of this observation was presented, and in particular it is unknown whether these patients were suffering from OA [28].

Apart from the detection of FAP-positive FLSs in RA and OA, substantial differences between both diseases regarding this cell type are apparent. First, FAP-expressing FLSs in RA synovial tissue are more frequent and predominantly localised in the lining layer. Second, FAP expression by RA FLSs is mainly by FLSs of the myofibroblastic phenotype. Third, RA joints show an accumulation of MMP-1 and MMP-13, in concert with the v3 and v7/8 splice variants of CD44 in the synovial lining layer. In contrast, osteoarthritic joints show minor expression of MMPs and CD44 variants. Because FAP expression is much more pronounced in RA tissue, this might be related to the degree of synovial inflammation, as has been already demonstrated for MMPs and FAP in collagen-induced arthritis (CIA). In mice, analysis of CIA gene expression profiles revealed a seven-fold increase in FAP, DPP IV, and MMP gene expression in inflamed paws as compared with non-inflamed paws [29,30]. Furthermore, DPP IV is a structural homologue to FAP, and seprase and FAP are known to build heteromeric complexes with DPP IV/CD26 to act coordinately in ECM breakdown [5].

The fact that FLSs in the rheumatoid synovium express FAP intensively does not allow definitive conclusions regarding the functional enzymatic role in ECM degradation, because substrate specificity of FAP remains unknown [31]. However, the probability of its contribution to exacerbating joint degeneration was recently discussed [32] and is supported by our results. Characterisation of the synovial lining layer in patients with RA revealed that FAP expression is accompanied by accumulation of other degradation markers that are predominantly found in this area. MMP-1 and MMP-13 as well as the v3 and v7/8 splice variants of CD44 are instrumental in ECM remodelling in malignancies and are also already known to be present in the synovial membranes of diseased joints [14-18]. In addition, fibroblasts of the myofibroblastic phenotype seem to be the major cell type expressing FAP in patients with RA. They are also an important source of other proteolytic enzymes and are generally thought to be responsible for ECM degradation [20,33,34]. A reliable marker for identifying this subpopulation is anti-SMA. Immunohistochemistry revealed the expression of anti-SMA by FAP-positive FLSs in the intimal lining layer. The expression signature characterises the area of FAP-expressing myofibroblastic cells as the centre of high-inflammation activity in the rheumatoid synovium and discriminates from Thy-1-positive non-proliferating fibroblasts in the synovial matrix [35].

The potential value of FAP as a therapeutic target in RA and OA is twofold. First, if the proteolytic activity of FAP contributes to ECM degradation, inhibition is an attractive goal. In this context, it needs to be noted that suppression of enzymatic activity resulted in potent antitumour effects and could augment antibody-dependent cell-mediated cytotoxicity in murine tumour models [36,37]. Second, FAP appears to be a valuable marker for the identification of FLSs with the highest proteolytic activity. The genetically stable and restricted expression of FAP [6] leads to the establishment of several preclinical strategies for tumour therapy [23,38,39]. The concept of anti-FAP antibody-based cancer immunotargeting has even been proven in a clinical Phase I dose-escalation study [28].

Despite the elucidation of FLSs as key players in joint inflammation and proteolytic enzymes like MMPs as markers responsible for ECM degradation, no anti-fibroblast-directed therapy is currently available. Approaches to inhibit the joint-destructive process in RA by elimination or inhibition of one proteolytic enzyme did not produce sufficient results in clinical trials, regardless of supportive *in vivo *results [40-42]. The difference between the targeting strategies of these approaches and FAP-specific targeting results from the substantial potential of FAP as a specific marker for synovial fibroblasts in RA. Therefore, targeting strategies could involve both inhibiting the role of the enzyme in tissue remodelling and focusing antibody-mediated cytotoxic activity on this synovial cell type to eradicate a central cellular mediator of the joint-destructive process [43].

## Conclusion

Expression of FAP by RA FLSs has not been described so far and highlights an implication in cartilage and bone destruction of arthritic joints. The specific expression pattern qualifies FAP as a therapeutic target for eradicating the destructive fibroblast-like synovial cell type.

## Abbreviations

CIA = collagen-induced arthritis; DPP IV = dipeptidylpeptidase IV; ECM = extracellular matrix; FAP = fibroblast activation protein; FLS = fibroblast-like synoviocyte; GADPH = glyceraldehyde-3-phosphate dehydrogenase; HPRT = hypoxanthine phosphoribosyltransferase; IgG = immunoglobulin G; MMP = matrix metalloproteinase; OA = osteoarthritis; RA = rheumatoid arthritis; SMA = smooth muscle actin.

## Competing interests

The authors declare that they have no competing interests.

## Authors' contributions

SB, MCJ, and CR helped to conceive, design, and coordinate the study and draft the manuscript. RD and FS helped to conceive the study and carried out synovial membrane preparations. AR coordinated human synovium collection and carried out immunohistochemistry and cell culture. EF performed the double-staining. AW and SK carried out preparation of cDNA, RT-PCR, and LightCycler PCR. SG and HD collected human synovial tissue from joint replacement surgery. UM-L helped to coordinate the study and draft the manuscript. All authors read and approved the final manuscript.
